# Rifampicin‐resistant RpoB S522L *Vibrio vulnificus* exhibits disturbed stress response and hypervirulence traits

**DOI:** 10.1002/mbo3.1379

**Published:** 2023-09-10

**Authors:** Laura Cutugno, Conor O'Byrne, Jan Pané‐Farré, Aoife Boyd

**Affiliations:** ^1^ School of Natural Sciences University of Galway Galway Ireland; ^2^ School of Biological and Chemical Sciences University of Galway Galway Ireland; ^3^ Centre for Synthetic Microbiology (SYNMIKRO) & Department of Chemistry Philipps‐University Marburg Marburg Germany

**Keywords:** rifampicin resistance, rpoB, stress response, *Vibrio vulnificus*, virulence

## Abstract

Rifampicin resistance, which is genetically linked to mutations in the RNA polymerase β‐subunit gene *rpoB*, has a global impact on bacterial transcription and cell physiology. Previously, we identified a substitution of serine 522 in RpoB (i.e., RpoB^S522L^) conferring rifampicin resistance to *Vibrio vulnificus*, a human food‐borne and wound‐infecting pathogen associated with a high mortality rate. Transcriptional and physiological analysis of *V. vulnificus* expressing RpoB^S522L^ showed increased basal transcription of stress‐related genes and global virulence regulators. Phenotypically these transcriptional changes manifest as disturbed osmo‐stress responses and toxin‐associated hypervirulence as shown by reduced hypoosmotic‐stress resistance and enhanced cytotoxicity of the RpoB^S522L^ strain. These results suggest that RpoB‐linked rifampicin resistance has a significant impact on *V. vulnificus* survival in the environment and during infection.

## Graphical abstract

Variant *rpoB* alleles affect *Vibrio vulnificus* stress responses and cytotoxicity. RpoB S522L mutants have decreased hypo‐osmolarity tolerance and survival. Rifampicin‐resistant RpoB S522L *Vibrio vulnificus* has a hypervirulent phenotype.

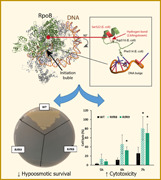

## 1 INTRODUCTION

Antibiotic resistance is of growing concern and poses a severe threat to human health and the global economy (WHO, 2014). Over the past decades, efforts have been increasingly made to understand the occurrence and dissemination of antibiotic resistance (Naylor et al., 2018; Zheng et al., 2021) and to find alternatives to classical antimicrobial treatments (Ghosh et al., 2019; Kumar et al., 2021). A consequence of antibiotic resistance is the pleiotropic effect that mutations conferring resistance can have on bacterial physiology and, consequently, the dissemination and evolution of antibiotic resistance (Gifford et al., 2018; Maharjan & Ferenci, 2017; Schenk & de Visser, 2013). In this work, attention is directed to the effects of a mutation conferring rifampicin resistance on stress responses and virulence in the human pathogen *Vibrio vulnificus. V. vulnificus* is a gram‐negative marine bacterium that causes severe gastroenteritis or septicemia through consumption of undercooked seafood, in particular oysters, or through wound infections via contact with contaminated seawater (Oliver 2005, 2015). It exhibits features of an opportunistic pathogen as successful infection is favored by pre‐existing health conditions, such as hemochromatosis (Bullen, 1991; Linkous & Oliver, 1999; Wright et al., 1981). Infection is fatal in approximately 50% of patients, as a result of septicemia and organ damage (Jones & Oliver, 2009).

Like most human pathogens, survival in the environment and the human host are key stages of the *V. vulnificus* infection process (Fang et al., 2016) along with the production of a range of toxins and exoenzymes (Miyoshi, 2013; Oliver et al., 1986; Pérez‐Reytor et al., 2018). The influence of mutations conferring rifampicin resistance on physiological fitness and virulence during infection has so far received little study for *V. vulnificus* (Cutugno et al., 2020). Antibiotic resistance, such as resistance to rifampicin, is often reported in both clinical and environmental isolates of *V. vulnificus* (Ottaviani et al., 2001) and other *Vibrio* spp. (Heng et al., 2017; Oh et al., 2011). Phenotypic characterization of rifampicin‐resistant *V. vulnificus* would enable prediction of their ability to thrive and establish themselves in the environment and to cause infections in humans.

Rifampicin is a broad‐spectrum antibiotic used in the treatment of bacterial infections (Alifano et al., 2015). It interferes with DNA transcription by binding to the β‐subunit (RpoB) of the bacterial RNA polymerase (RNAP) (di Mauro et al., 1969). Rifampicin resistance emerged shortly after the introduction of this antibiotic (Manten & Van Wijngaarden, 1969) and is usually caused by mutations that alter residues of the rifampicin binding site in RpoB, resulting in decreased affinity for rifampicin (Ezekiel & Hutchins, 1968; Jin & Gross, 1988; Lisitsyn et al., 1984; Ovchinnikov et al., 1981, 1983). Alternatively, ADP‐ribosylation of rifampicin can inactivate the drug (Baysarowich et al., 2008). Secondary effects of mutations in *rpoB* have been characterized in several bacteria (Alifano et al., 2015; Billington et al., 1999; Colicchio et al., 2015; Jin & Gross, 1989; Jin et al., 1988a; 1988b; 1988c; Wichelhaus et al., 2002) and have only recently been investigated in *Vibrio* (Cutugno et al., 2020).

In the present work, the focus was on two spontaneous rifampicin‐resistant *V. vulnificus* strains carrying a serine‐to‐leucine amino acid exchange at residue 522 in RpoB (RpoB^S522L^). This amino acid exchange is of particular significance because it has previously been shown to negatively impact the growth of *V. vulnificus*, while wild‐type levels of tolerance to ethanol, hyper‐osmotic, and acidic stress conditions were maintained (Cutugno et al., 2020). Furthermore, substitutions in the same amino acid in *Escherichia coli* RpoB are associated with a stringent‐like phenotype (Alifano et al., 2015; Murphy & Cashel, 2003).

In this study, we show that transcription of specific sets of stress and virulence genes is significantly altered in *V. vulnificus* expressing RpoB^S522L^, thereby leading to changes in stress survival and cytotoxicity, and suggest that RpoB‐linked rifampicin resistance impacts the ability of the organism to thrive in the environment and to infect humans.

## 2 MATERIALS AND METHODS

### 2.1 Strains and growth conditions

The bacterial strains used in this study were *V. vulnificus strain* CMCP6 (Kim et al., 2003) and two previously isolated rifampicin‐resistant derivatives of this strain, Rif^R^8 and Rif^R^9, which possess the *rpoB* S522L allele (Cutugno et al., 2020). Whole genome sequencing of Rif^R^8 and Rif^R^9 revealed the following nonsynomous mutations: RifR8—*rpoB* S522L, *mviN* A158V, *recA* G179S, *VV1_2631* H340Y, *VV2_0149* R334H and RifR9—*rpoB* S522L, *VV1_2631* H340Y, *VV2_0149* R334H, *VV2_0774* W195* (Cutugno et al., 2020). In this study, we analyzed both strains in phenotypic assays to exclude effects due to unique secondary mutations in each genome.

Strains were cultured in lysogeny broth (LB) medium (NaCl 10 g/L, tryptone 10 g/L, yeast extract 5 g/L) supplemented with additional 15 g/L NaCl (LBN, total 0.4 M NaCl ) or chemically defined medium (CDM) (10 mM Na_2_HPO_4_, 10 mM KH_2_PO_4_, 0.81 mM MgSO_4_ ∙ 7H_2_O, 9.35 mM NH_4_Cl, 427 mM (25 g/L) NaCl, 7.5 mM α‐D(+)‐glucose, 0.75 μM FeCl_3_) at pH 6.6. Overnight cultures were grown at 30°C or 37°C in 2 mL medium in 15 mL bacterial culture tubes or 20 mL medium in 250 mL flasks. All chemicals and reagents were sourced from Sigma–Aldrich unless otherwise indicated.

### 2.2 In silico evaluation of the S522L mutation

To evaluate the potential structural consequences of the S522L amino acid substitution in *V. vulnificus* RpoB we interpreted the *E. coli* RNA polymerase holoenzyme structures (PDB‐ID: 7KHB) with bound ppGpp (PDB‐ID:7KHI). Structures were downloaded from the PDB database (rcsb.org) and visualized and evaluated in Pymol (https://pymol.org/2/).

### 2.3 Growth characterization

Growth was assessed at 30°C in LBN or CDM and at 37°C in Dulbecco's modified eagle medium (DMEM). For the growth characterization in CDM and LBN, the strains were grown overnight in 20 mL LBN at 30°C. In initial experiments, overnight cultures were serially diluted and plated on LBN agar, and following overnight incubation the number of colony‐forming units was counted to verify equitable numbers of viable bacteria in the culture of each strain. Bacterial cultures were then washed and diluted in 30 mL LBN or 30 mL CDM to a final OD_600_ of 0.05 in 250 mL flasks. The flasks were incubated at 30°C with 150 rpm agitation. OD_600_ was measured every 60 min for the first 10 h and lastly at 24 h. The specific growth rate (μ) between two time points (t1 and t2) taken during the exponential phase was calculated using the formula *μ* = ln2/*g*, where *g* is the doubling time. At least three biological replicates were used for each strain. To study growth in DMEM, the strains were analyzed in microtiter plates. Overnight cultures were grown in 2 mL LBN at 37°C and, after a washing step, diluted in DMEM at an initial OD_600_ of 0.01. Three biological replicates were used for each strain and each of them was assessed in two technical replicates. The plates were incubated statically in an LT‐5000 MS microtiter plate reader at 37°C and the OD_600_ was measured, following 5 s of shaking, every 15 min for 48 h.

To assess the minimum required concentration (MRC) of NaCl, a microtiter plate was set up with two‐fold dilutions of NaCl in Mueller–Hinton (MH) broth, from 0.4 to 0.03 M, and broth without additional salts was used as a blank. MH (peptone 17.5 g/L, meat extract 2 g/L, starch 1.5 g/L) was selected as a growth medium in which *V. vulnificus* CMCP6 would grow without NaCl supplementation. The osmolality of MH broth is 0.3 Osm/kg. CMCP6 failed to grow on tryptone 10 g/L, yeast extract 5 g/L (0.1 Osm/kg). All the strains were cultured overnight in LBN at 37°C and, after a washing step, inoculated in the microtitre plate to an initial OD_600_ of 5 × 10^−4^. Three biological replicates were used for each strain and each of them was assessed in two technical replicates. The plates were incubated for 24 h at 30°C and the final OD_595_ was measured with a Sunrise microtiter plate reader. The viability of the bacteria at the end of the experiment was assessed by determining the number of cfu/mL. The bacteria were serially diluted in 10‐fold steps and 10 µL was spotted in triplicate onto LBN agar. After 24 h incubation at 37°C the number of colonies was counted and the cfu/mL calculated.

To test the growth on solid media in the absence of NaCl, the Clinical and Laboratory Standards Institute (CLSI) M45 protocol was applied (Clinical and Laboratory Standards Institute [CLSI], 2015). Briefly, the strains were grown overnight on LBN agar at 37°C. Several single colonies were selected for resuspension in 0.85% NaCl to reach OD_600_ 0.1. A sterile cotton swab was dipped into the cell suspension and streaked on MH agar. MH + 0.4 M NaCl agar was used as a control. Plates were allowed to dry at room temperature and incubated at 37°C for 16 h.

### 2.4 RNA extraction and qRT‐PCR gene transcription analysis

Once the phenotypic traits common to both Rif^R^8 and Rif^R^9 were determined, the associated transcriptional analysis was performed solely on the Rif^R^9 strain. For RNA extraction, cell pellets were harvested from overnight cultures in 2 mL LBN at 37°C or exponential (OD_600_ 0.5) and stationary (OD_600_ 2.5) phase during growth in 30 mL LBN at 30°C. Two volumes of RNAprotect (Qiagen) were mixed with one volume of bacterial culture containing approximately 0.5 OD_600_ of cells and processed following the manufacturer's instructions. To lyse the bacterial cells, the pellet was re‐suspended in 200 µL TE buffer (30 mM Tris‐HCl, 1 mM EDTA, pH 8.0),15 mg/mL lysozyme,1 mg/mL Proteinase K and incubated at room temperature for 10 min. The lysate was then used for RNA extraction with an RNeasy Mini Kit (Qiagen) according to the manufacturer's protocol. RNA was eluted in 50 µL RNase‐free water and a rigorous DNase treatment was performed using the TURBO DNA‐free kit (Life Technologies), according to the manufacturer's instructions. RNA integrity was verified on a 1.5% agarose gel and concentration and quality were assessed using a NanoDrop spectrophotometer. Only the samples with a 260/280 ratio ≥1.9 were used for the qRT‐PCR protocol. The Transcriptor First Strand cDNA Synthesis Kit (Roche) and the Random Hexamer Primer provided by the manufacturer, were used to synthesize cDNA from 1 µg total RNA. The RT reaction was carried out as follows: 10 min at 25°C followed by 60 min at 50°C. Primer‐BLAST (Ye et al., 2012) was used to design the primers for the qPCR reactions (Table 1) and, before use, the primer specificity and efficiency were calculated. Two microliters of a 1:10 dilution of the cDNA were used in a 10 µL reaction to study transcription levels of the several gene targets through qPCR, using the SYBR Green I Master (Roche) and the LightCycler 480 (Roche). Two technical replicates were utilized to test each reaction. The transcription of each gene was normalized using *tuf* (elongation factor Tu) as endogenous control, similar to previous transcription studies (Nowakowska & Oliver, 2013). Fold changes in transcription levels were calculated using the Pfaffl method (Pfaffl, 2001).

**Table 1:** Primers for quantitative real‐time PCR (qRT‐PCR) of stress and virulence target genes in *Vibrio vulnificus*.

Gene	Sequence (5′–3′)
*tuf* (VV1_1203)	Forward: AAGTTTACGGCGGTGCTGCT Reverse: CGTAGTGGCGAGCTGGAGTG
*rmf* (VV1_2630)	Forward: ACGAACGAGCGTCCATCTGT Reverse: TCCCAAGGCTACAAGGCAGG
*fusA* (VV1_1338)	Forward: TGGCATTCAAGAAGGGCGCA Reverse: GCGACGGTTCAAGTCACCCA
*relA* (VV1_1575)	Forward: GTGGGTGTTGGCAGTGGTGA Reverse: GCGGCTTTATTGCCCGCTTC
*putA* (VV2_1118)	Forward: CACTGGCCCCACATCGGTTT Reverse: GAGCAGGTGGTGCGTGATGT
*rpoS* (VV1_1588)	Forward: CCAGAGCGTGGTTTCCGCTT Reverse: GAGAAAGCTCACGCGCCGTA
*toxR* (VV1_0190)	Forward: ATGCTGGCACGTCAACAAAGATGG Reverse: TGGTGAGCAAGACAACGCAAAGTG
*ompU* (VV1_1931)	Forward: TAGGTGGCAAGTTTGGTGAAGTGA Reverse: GCTTAGTGCATCGAATTGGCCTTT
*vvhA* (VV2_0404)	Forward: CGGTACAATCGGCAACGTCA Reverse: GGCGAATGGACCAATGTAAGTGC
*vvpE* (VV2_0974)	Forward: CCTGAGCGTCCTTCTGTCGC Reverse: TCCGTCTCCGATAGTGCCGT
*rtxA* (VV2_0479)	Forward: ACGCCCACCAAAACCGTCAT Reverse: ATTGCTTCTGCTGGCGTGGT

### 2.5 Cytotoxicity assay

To study cytotoxicity, HeLa cell lysis was assessed following co‐incubation with bacterial cells or bacterial culture supernatant. In both cases, HeLa cells were seeded in 48‐well plates with DMEM + 10% fetal bovine serum (FBS) + 1% pen/strep. Before co‐incubation, the HeLa cells were washed twice with DMEM + 10% FBS without antibiotics and allowed to recover for 30 min before adding the bacterial cells or the bacterial supernatant. Bacterial cultures were grown overnight in LBN broth at 37°C. To test the cytotoxicity of the supernatant, 1 mL overnight culture was centrifuged at 13,000 rpm for 2 min and the supernatant was filtered using a 0.22 µM filter. One hundred microliters filtered supernatant was added to the well containing the HeLa cells in 900 µL DMEM. For the co‐incubation experiment, the overnight cultures were diluted in fresh LBN broth to a final OD_600_ of 0.05 and grown to the mid‐log phase (OD_600_ between 0.4 and 0.6). When the optimum OD_600_ was reached, the bacterial cultures were first diluted to OD_600_ 0.01 in PBS and then to a final OD_600_ of 0.001 (equivalent to 1 × 10^6^ colony forming units [cfu]/mL) in the DMEM containing the HeLa cells to achieve a multiplicity of infection (MOI) of 10. The plates were not centrifuged, rather the bacteria were allowed to settle onto the cells. Following incubation at 37°C for various periods, HeLa cell lysis was determined, via Lactate Dehydrogenase (LDH) quantification, using CytoTox 96 Nonradioactive Cytotoxicity Assay (Promega), according to the manufacturer's protocol. The positive control (100% lysis) was a sample of HeLa cells treated with 1X Lysis buffer (Promega). HeLa cells in the absence of bacteria were used as negative control (0% lysis). In the case of supernatant cytotoxicity, 100 µL sterile LBN was added to the HeLa cells as a negative control (0% lysis) to exclude the cytotoxic effects of the media itself. For each strain, three biological replicates, each assessed in technical triplicates, were analyzed in at least two independent experiments. During co‐incubation experiments, images of the plates were acquired with a Leica DFC420 C microscope at x40 magnification.

## 3 RESULTS

### 3.1 The S522L substitution maps to a region in RpoB critical for RNA polymerase function

Serine 522 is located in rifampicin cluster I of the RNA polymerase β‐subunit (Alifano et al., 2015; Cutugno et al., 2020) where the majority of amino acid substitutions conferring rifampicin resistance occur (Alifano et al., 2015) but it is not directly involved in binding rifampicin (Alifano et al., 2015). First, we wanted to understand the structural consequences of the exchange of serine 522 to leucine on RpoB in the context of the RNA polymerase holoenzyme (RNAP). For this purpose, we employed a high‐resolution structure of *E. coli Ec*RpoB bound within *Ec*RNAP to infer possible consequences of the serine 522 to leucine substitution in RpoB. *Vv*RpoB is highly conserved with its counterpart in *E. coli*, displaying 85% sequence identity and 92% conservation at the amino acid level.

**Figure 1:**
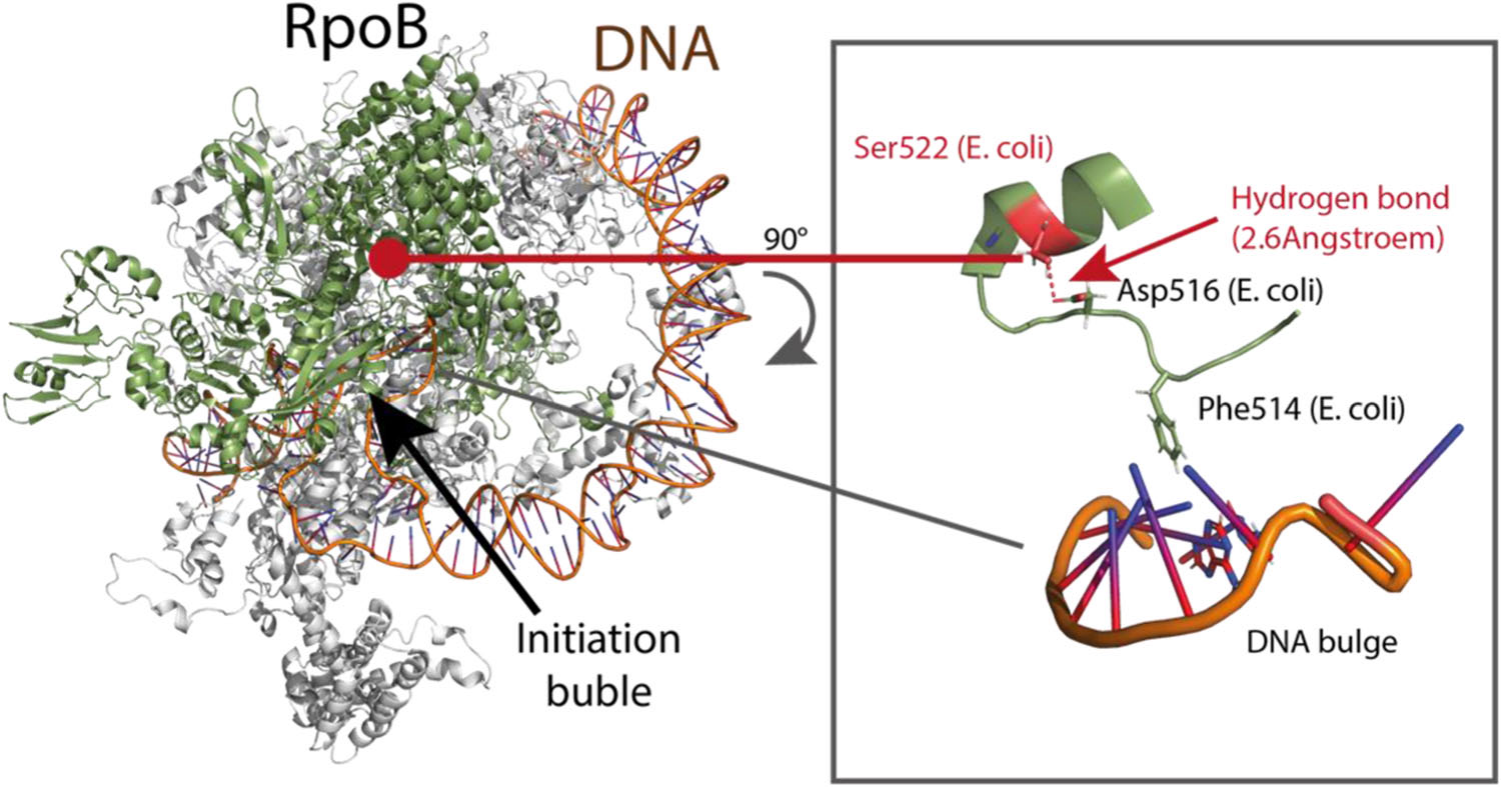
The RpoB^S522L^ variant potentially affects geometry of the transcription initiation bubble. The *Escherichia coli* RNA polymerase *rrnBP1* promoter open complex (PDB‐ID: 7KHB) is shown on the left. The zoom inset on the right‐hand side shows RpoB^S522^ forming a hydrogen bond with RpoB^D516^. Residue D516 is located within the loop region of RpoB that interacts with the DNA bulge in the open complex.

In the *E. coli* structure, serine 522 forms a hydrogen bond with aspartate 516 of RpoB, and this bond appears to be critical for the productive action of rifampicin at the RpoB‐RNAP complex. It has been shown that the substitution of aspartate 516 confers high levels of rifampicin resistance and it is frequently detected in clinical isolates of rifampicin‐resistant bacteria (Andre et al., 2017). The serine 522 to leucine substitution of RpoB would disrupt the hydrogen bond between serine 522 and aspartate 516. Therefore, we speculate that the serine‐to‐leucine substitution at residue 522 has the potential to alter the geometry of the active site of RpoB, and thus to change the transcriptional activity of RNAP (Figure 1). Interestingly, in *Streptomyces coelicolor* rifampicin resistance mutations located in rifampicin cluster I of *rpoB* restore the ppGpp‐dependent production of the antibiotic actinorhodin in *relA* and *rplK* strains that fail to induce a stringent response (Xu et al., 2002). Among the amino acid substitutions identified in the *S. coelicolor* study was *Sc*RpoB^S423T^, which corresponds to serine 522 in the *Vv*RpoB protein. This observation suggests that the rifampicin resistance conferred by *Vv*RpoB^S522L^ could also functionally mimic the modifications induced in the RNA polymerase by binding of ppGpp (Xu et al., 2002). Although the exact physiological consequences of the RpoB^S522L^ substitution are difficult to predict for *V. vulnificus*, it seems possible that it could have an impact on gene transcription and growth of *V. vulnificus* in response to stress conditions, which merited further investigation.

### 3.2 The S522L substitution causes a growth defect in rich media, but not in chemically defined media

**Figure 2:**
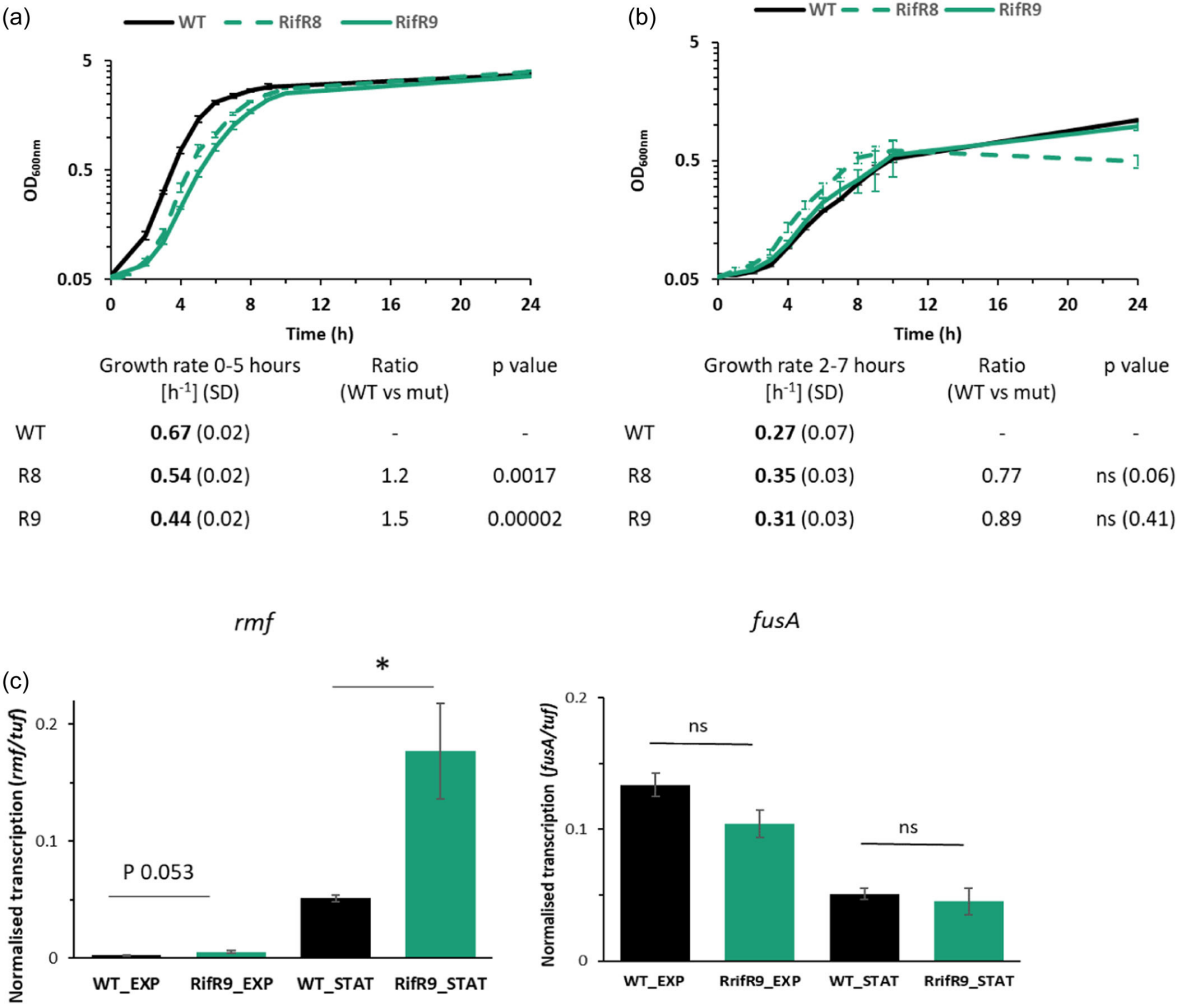
Growth profile of RpoB^S522L^ *Vibrio vulnificus*. Growth curves of *V. vulnificus* CMCP6 wild type (WT) (black), Rif^R^8 (dashed green), and Rif^R^9 (solid green) in (a) LBN and (b) chemically defined medium (CDM). The data points are the mean values of at least five biological replicates. The growth rates of each strain and the ratio between mutant and wild‐type growth rates are reported below the graph. Student's *t*‐test compares the growth rate of each RpoB^S522L^ strain to the wild type. (c) *rmf* and *fusA* gene transcription in *V. vulnificus* CMCP6 wild type and Rif^R^9 during exponential (EXP) and stationary (STAT) growth in LBN. Normalized gene transcription values, using *tuf* as the reference, are reported. Values presented are the mean ± SD of three biological replicates. Student's *t*‐test was performed comparing transcription in the wild type and the mutant (*p ≤ 0.05; ns,*p* > 0.05).

We had previously demonstrated that two Rif^R^ *V. vulnificus* strains (Rif^R^8 and Rif^R^9), carrying the S522L amino acid substitution in RpoB, have a growth defect in LBN medium (Cutugno et al., 2020). In this work, the wild‐type CMCP6 and the RpoB^S522L^ *V. vulnificus* strains were assessed for growth in both nutrient‐rich and nutrient‐limited conditions. To this end, the wild type and Rif^R^8 and Rif^R^9 were grown at 30°C in 30 mL LBN or CDM (Figure 2). The rifampicin‐resistant strains showed a growth defect in LBN medium compared to the wild type, with a slower growth rate during exponential growth, though eventually achieving the same biomass accumulation in stationary phase. This difference was not observed in CDM (Figure 2). A similar growth phenotype has been described for other bacterial species with mutations in RNAP that cause a stringent‐like phenotype, mimicking the presence of (p)ppGpp (Conrad et al., 2010; Murphy & Cashel, 2003).

To investigate whether RpoB^S522L^ *V. vulnificus* influenced the stringent response, we investigated the transcription of *rmf*, encoding ribosome modulation factor, and *fusA*, encoding translation elongation factor G (EF‐G), which are up‐ and downregulated by (p)ppGpp in *E. coli*, respectively (Izutsu et al., 2001; Zhang et al., 2016). Gene transcription analysis of wild‐type *V. vulnificus* revealed reduced transcription of the *fusA* gene in stationary phase compared to exponentially growing bacteria in LBN, as expected due to the general downregulation of the translation machinery upon induction of the stringent response when bacteria enter stationary phase (Pletnev et al., 2015). Conversely, *rmf* transcription was increased in the stationary phase, in line with previous studies (Yamagishi et al., 1993). While there was no significant difference in *fusA* transcription between the wild type and the Rif^R^9 mutant, there was a 3.5‐fold higher *rmf* transcription during stationary phase in Rif^R^9 as compared to CMCP6 (Figure 2c). These data suggested that the RpoB^S522L^ variant may influence the stringent response.

### 3.3 The RpoB^S522L^ variant alters the hypoosmotic stress response of *V. vulnificus* and the transcriptional profile of stress‐related genes

**Figure 3:**
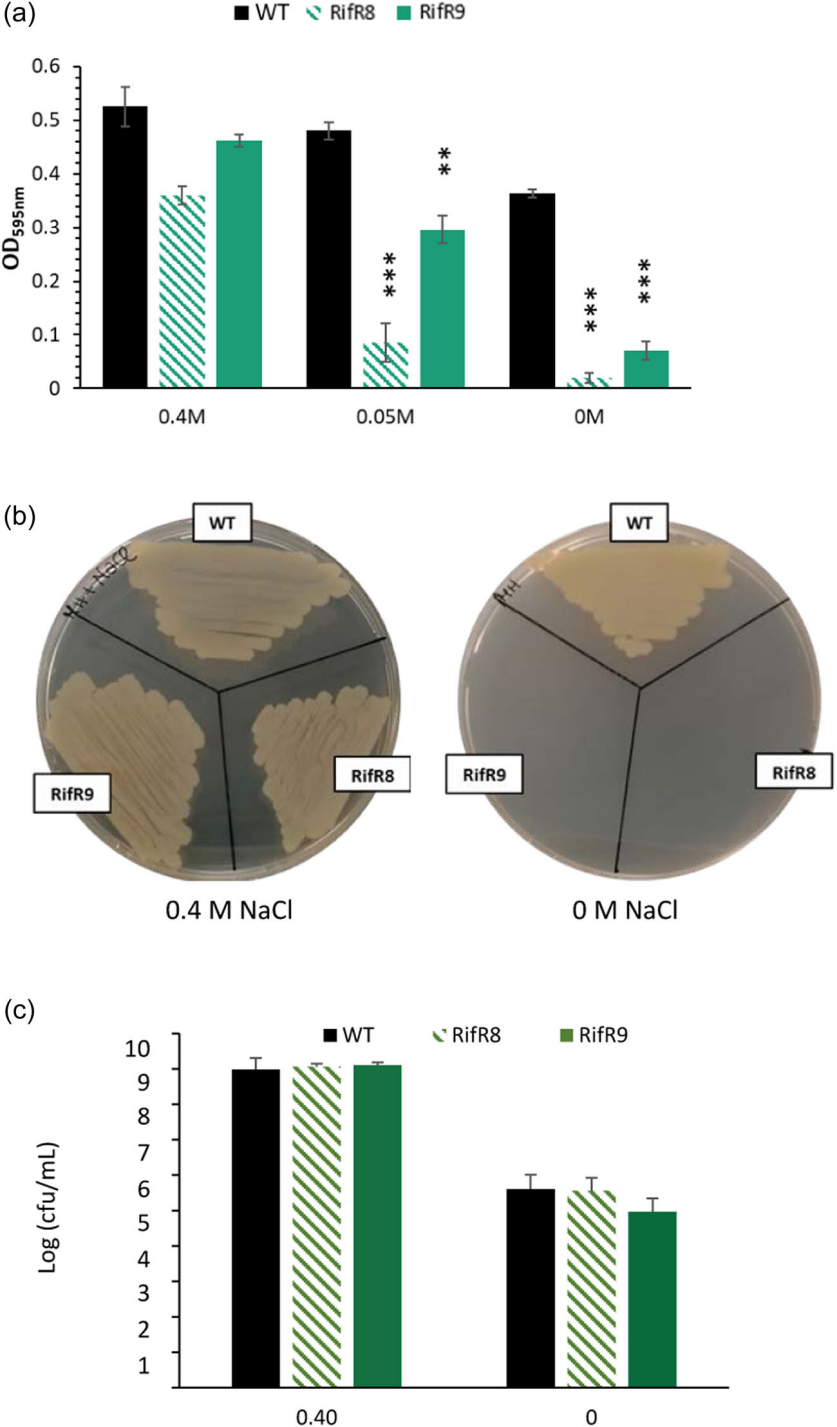
Impaired hypoosmotic stress response of RpoB^S522L^ *Vibrio vulnificus*. (a) Growth of *V. vulnificus* wild type (black) and RpoB^S522L^ strains (green) in Mueller–Hinton (MH) broth supplemented with 0.4, 0.05, or 0 M NaCl was measured via OD_595_ after 24 h culture. (b) Growth of RpoB^S522L^ and wild‐type *V. vulnificus* on MH agar in the absence (B.1) and presence (B.2) of 0.4 M NaCl after 24 h incubation. (c) Viability of wild type (black) and RpoB^S522L^ (green) *V. vulnificus* in MH broth supplemented with 0.4 or 0 M NaCl was measured via cfu after 24 h culture. Values reported are the mean ± SD of three biological replicates. Student's *t*‐test compared OD_595_ values for RpoB^S522L^ *V. vulnificus* with the wild type. **p ≤ 0.01; ***p ≤ 0.001.

**Figure A1:**
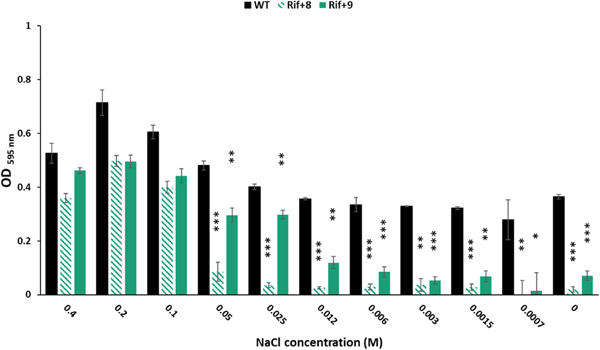
Complete NaCl minimum required concentration (MRC) profile of *Vibrio vulnificus* CMCP6 wild‐type (black) and S522L mutants (green) in Mueller–Hinton media. Growth at NaCl concentrations between 0.4 and 0 M was assessed in Mueller–Hinton broth. OD595 was measured after 24 h incubation at 30°C. Three biological replicates for each strain were tested and the reported values are the mean of the three replicates. Student's *t*‐test was performed comparing S522L strains with the wild‐type and *p*‐values are shown (**<0.01; ***<0.001).

Coastal and intertidal bacteria, such as *Vibrio*, must adapt to changing seawater salinities caused by daily tides and weather conditions (Jones et al., 2008; León Robles et al., 2013; Randa et al., 2004). Previous work in *E. coli* has shown that certain *rpoB* variants affect osmotic stress responses (Xiao et al., 2017). We recently reported that *V. vulnificus* strains with RpoB^S522L^ have a similar hyperosmotic stress response to that of the wild type (Cutugno et al., 2020). To further investigate the response of the RpoB^S522L^ strain to osmotic stress, hypoosmotic stress tolerance was analyzed in MH media which allows the growth of *Vibrio* spp. in the absence of supplementary NaCl (CLSI, 2015). MRC broth experiments revealed that both S522L mutants Rif^R^8 and Rif^R^9 reached a significantly lower optical density than the wild type after 24 h culture at low concentrations of NaCl (≤50 mM), while NaCl concentration had little influence on OD_600_ measurements of the wild type (Figure 3a and Appendix: Figure A1). When growth was assessed on MH agar, the wild type grew equally well with or without NaCl supplementation, while RpoB^S522L^ strains showed a clear dependence on NaCl supplementation (Figure 3b). Similar numbers of viable bacteria (9.7–12.9 × 10^8^ cfu/mL) were recovered for all three strains following 24 h culture in MH broth supplemented with 0.4 M NaCl (Figure 3c). In MH broth, the cfu/mL values for the 3 strains were much lower (9.3–40.6 × 10^4^ cfu/mL) and similar to the concentration of the starting inoculum (5 × 10^5^ cfu/mL). The low viability of the wild type was unexpected given its OD_600_ value and its strong visible growth on MH agar.

**Figure 4:**
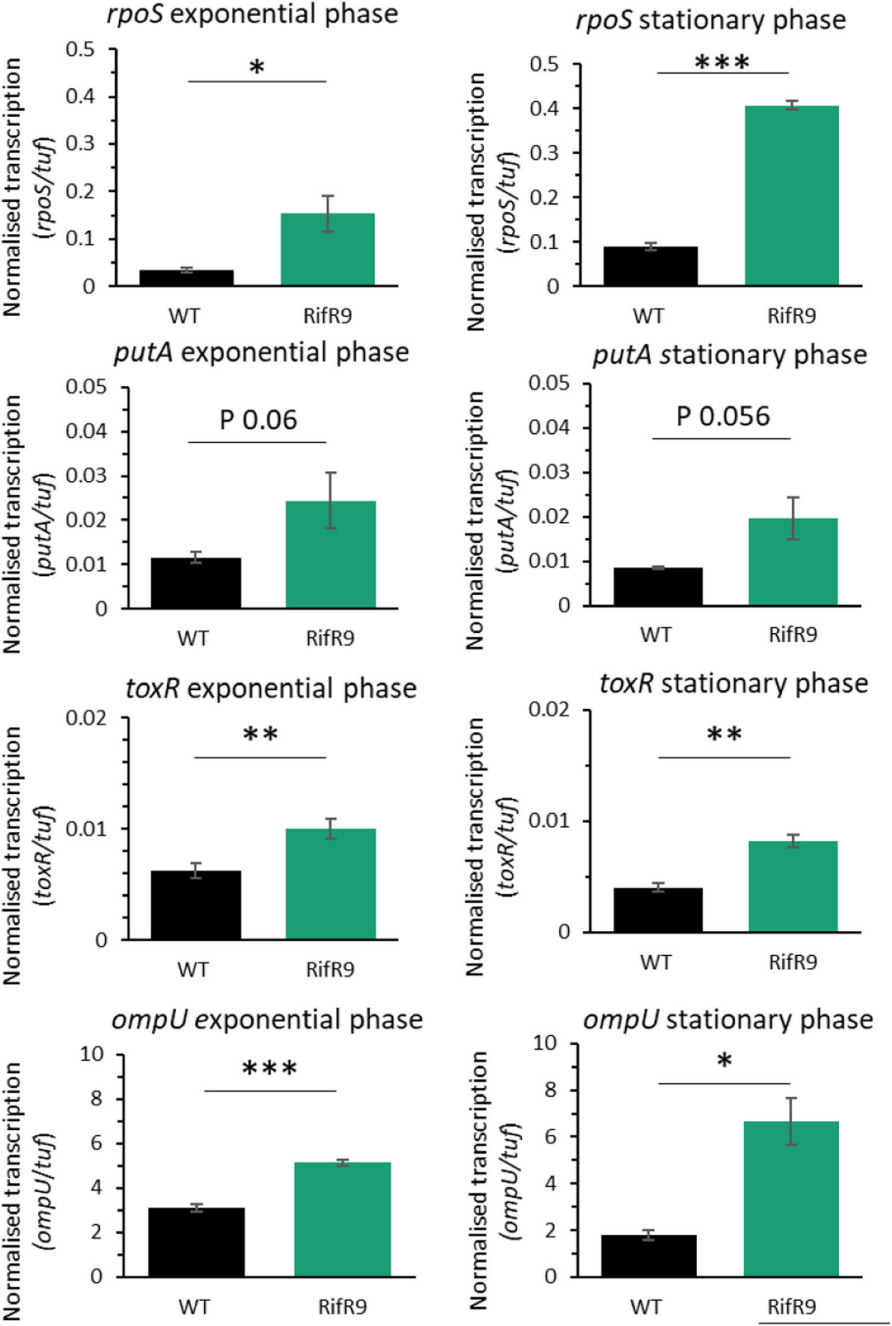
Increased stress‐related gene transcription in RpoB^S522L^ *Vibrio vulnificus*. Transcription of four genes (*rpoS, putA, toxR*, and *ompU*) in *V. vulnificus* wild type (black) and Rif^R^9 (green) at exponential and stationary growth phase in LBN, in the absence of stress, was analyzed. Normalized gene transcription values, using *tuf* as the reference, are reported as the mean ± SD of three biological replicates. Student's *t*‐test compared transcription in the wild type and in the mutant. *p ≤ 0.05; **p ≤ 0.01; ***p ≤ 0.001.

Hyper‐ and hypo‐osmotic stresses pose very different physiological challenges to the bacteria and the two types of response are generally opposing, with compatible solute synthesis and influx pumps used in response to high salt concentrations, while water influx, mechanosensory channels and transient pores are involved in the response to osmotic downshift (Gregory & Boyd, 2021; Rao et al., 2013). To probe if the hypoosmotic stress‐sensitive phenotype of the Rif^R^9 strain was correlated with a general dysregulation of the osmotic stress response, transcription of *putA* was analyzed in nonstressed cells during growth in LBN media. *putA* encodes proline dehydrogenase, which converts proline into glutamate—the major osmolyte required during hyperosmotic stress adaptation of *V. vulnificus* (Lee et al., 2003). *putA* transcription showed a trend, albeit not quite reaching statistical significance, towards higher levels in the Rif^R^9 strain than the wild type during exponential growth and in stationary phase (Figure 4). These results suggest that *V. vulnificus* expressing RpoB^S522L^ is characterized by an increased basal level of stress gene transcription.

### 3.4 The RpoB^S522L^ variant increases cytotoxicity through increased transcription of *vvhA* hemolysin

RpoS is a global regulator of stress responses (REF). Additionally, RpoS works together with ToxR to regulate virulence, including toxin and exoenzyme production, through mediating cross‐talk between stress response and virulence regulatory pathways (Chowdhury et al., 1996). ToxR is a global virulence regulator that senses cyclic dipeptides and is a component of the signaling network that stabilizes *rpoS* mRNA (Kim et al., 2018). Transcription of *rpoS*, *toxR*, and one of the latter's downstream effectors, *ompU*, was analyzed. These analyses showed increased transcription of *rpoS, toxR, and ompU* in the Rif^R^9 strain compared to the wild type during exponential growth and stationary phase in LBN (Figure 4). The increased *rpoS* transcription provides additional evidence for the increased basal level of stress gene transcription associated with *V. vulnificus* expressing RpoB^S522L^.

**Figure 5:**
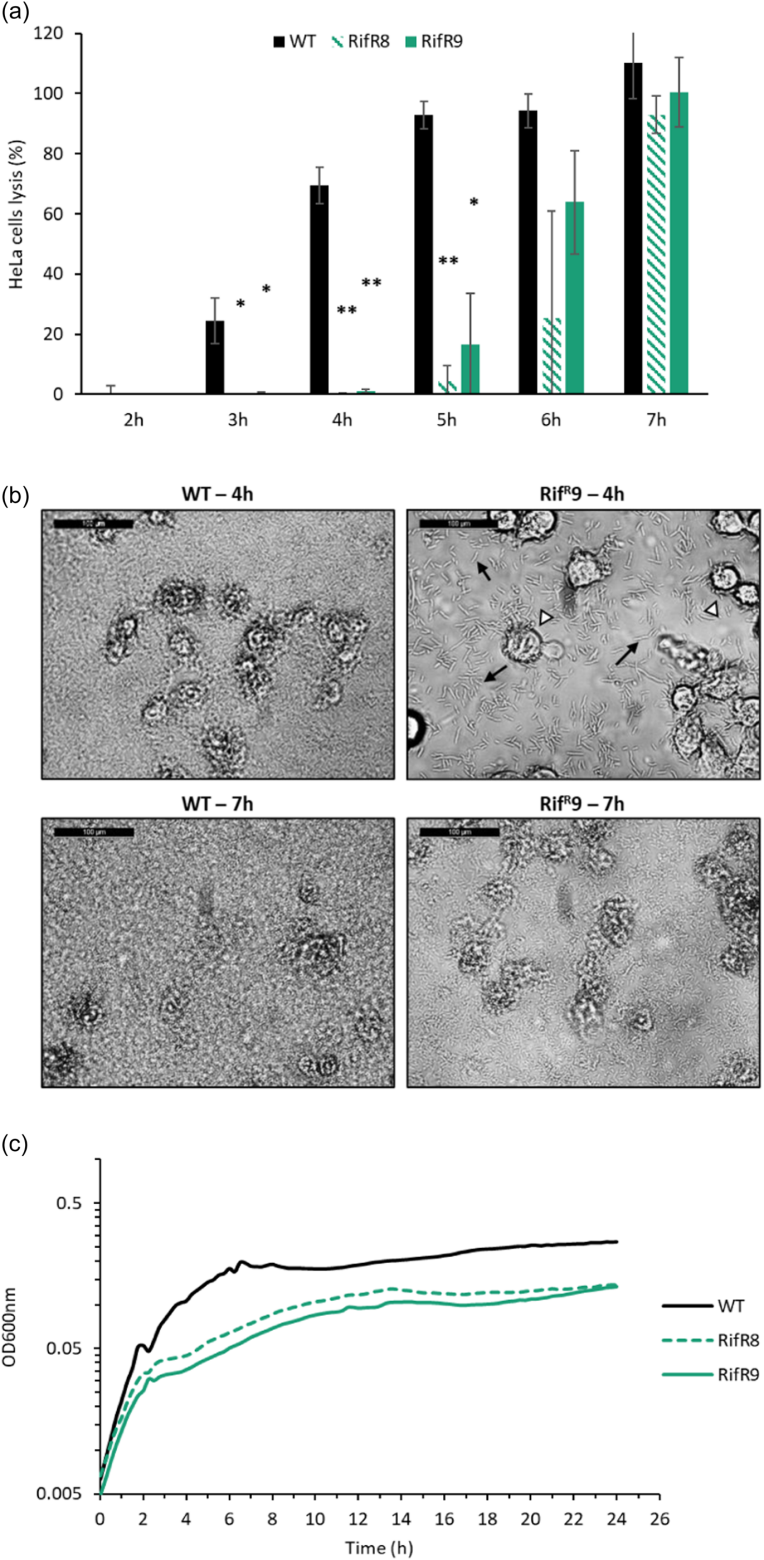
Cytotoxicity of wild type and RpoB^S522L^ *Vibrio vulnificus* in coculture conditions. (a) Coincubation of bacteria with HeLa cells and LDH quantification. The percentage of lysed HeLa cells, compared to a 100% lysis control, was calculated through LDH quantification at each hour after the addition of the wild type and RpoB^S522L^ *V. vulnificus* inoculum. The plates were not centrifuged, rather the bacteria were allowed to settle onto the cells. Reported values are the mean ± SD of three biological replicates. Student's *t*‐test compared the wild type and the variants. *p ≤ 0.05; **p ≤ 0.01. (b) Light phase microscopic images of coculture samples after 4 and 7 h of coincubation to demonstrate bacterial cell density. Bacterial cells or groups of bacterial cells are indicated by black arrows. HeLa cells are indicated by white arrowheads. (c) Growth curve of *V. vulnificus* CMCP6 wild type (black), Rif^R^8 (dashed green), and Rif^R^9 (solid green) in Dulbecco's modified eagle medium (DMEM). Data values are the mean of three biological replicates.

**Figure A2:**
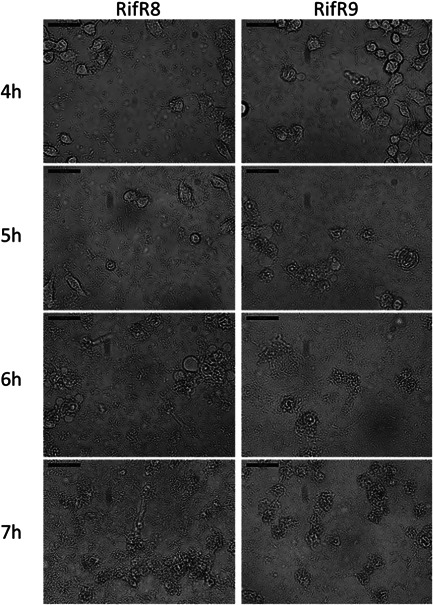
Cytotoxicity and viability of RpoBS522L *Vibrio vulnificus* in coculture conditions. Light phase microscopic images after 4, 5, 6, and 7 h of coincubation of RifR8 and RifR9 bacteria with HeLa cells.

RpoS upregulates the production of both the RTX toxin and the VvpE elastase (Guo et al., 2018; Hülsmann et al., 2003) while ToxR is a positive regulator of VvhA hemolysin production (Lee et al., 2000). To examine the effects of the S522L substitution on virulence, the cytotoxicity of wild type and RpoB^S522L^ *V. vulnificus* was examined. Bacteria were co‐incubated with HeLa cells in DMEM and cell lysis was subsequently evaluated through LDH quantification (Matlawska‐Wasowska et al., 2010). The Rif^R^8 and the Rif^R^9 strains displayed delayed cytotoxicity compared to the wild type, as demonstrated by the additional 2–3 h co‐incubation time needed to cause maximum eukaryotic cell lysis (Figure 5a). Although there were identical numbers of bacteria in the inoculum (1 × 10^6^ cfu/mL), visualization of the co‐incubated cells at later time points showed a greater abundance of wild‐type bacteria than of RpoB^S522L^ *V. vulnificus* (Figure 5b). This is particularly evident at the 4 h time point where it is possible to distinguish individual RpoB^S522L^ bacteria, whereas the wild type has increased in density to such an extent that the bacteria appear confluent. The continued viability and replication of both Rif^R^8 and Rif^R^9 is shown by their increase in bacterial density between 4 and 7 h (Appendix: Figure A2) and by the increase at 5 h in the number of viable bacteria to 2.6 × 10^7^ and 5.0 × 10^7^ cfu/mL, respectively. In contrast, the wild type reached 3.0 × 10^8^ cfu/mL at the same time point. Moreover, in the latter sample, HeLa cells display a greater degree of morphological disruption. To investigate whether differential bacterial growth rates in DMEM eukaryotic cell culture media could be the cause of the delayed cytotoxicity kinetics, the growth of the three strains was analyzed and revealed a major growth defect associated with the RpoB^S522L^ variant in this medium (Figure 5c).

**Figure 6:**
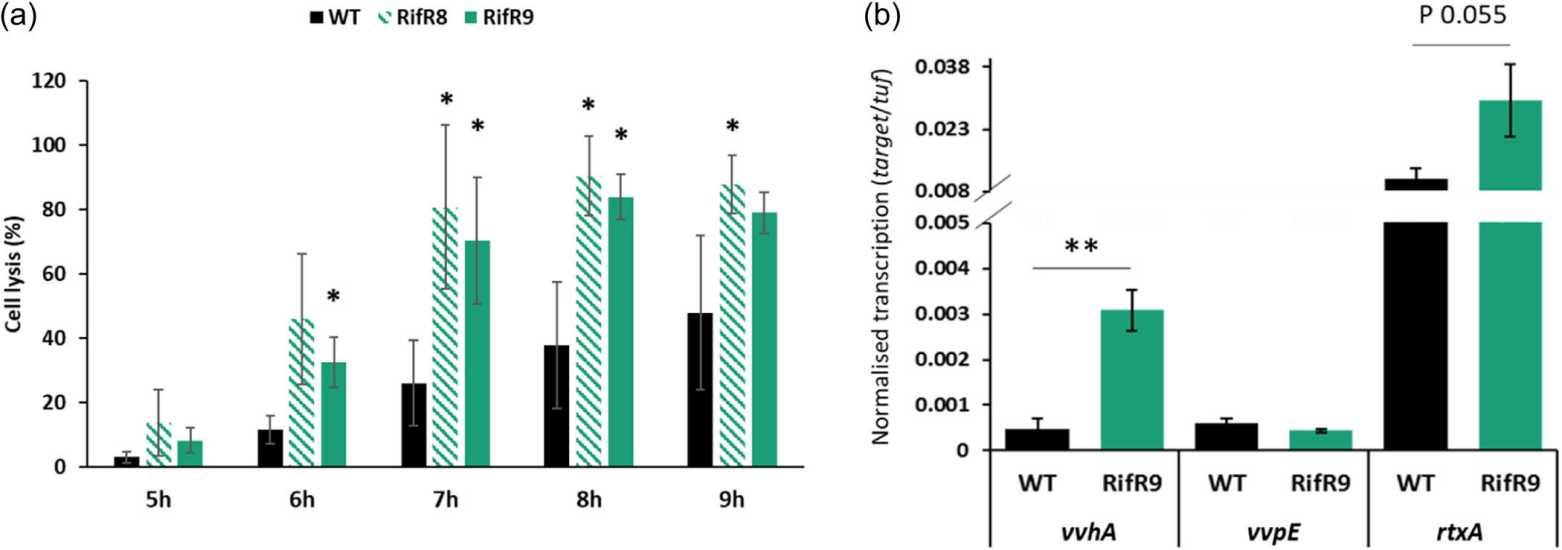
Elevated contact‐independent cytotoxicity of RpoB^S522L^ *Vibrio vulnificus*. (a) The cell‐free supernatant of overnight cultures of *V. vulnificus* wild type (black), Rif^R^8 (dashed green), and Rif^R^9 (solid green) grown in LBN was incubated with HeLa cells. The percentage of lysed HeLa cells, compared to the 100% lysis control, was calculated through LDH quantification each hour between 5 and 9 h after the addition of the supernatant. Reported values are the mean ± SD of three biological replicates. Student's *t*‐test compared the wild type and the variants. *p ≤ 0.05. (b) Toxin and exoenzyme gene transcription in *V. vulnificus* wild type (black) and Rif^R^9 (green) in overnight pre‐cultures in LBN. The transcription of *vvhA, vvpE*, and *rtxA* was analyzed, and normalized gene transcription values, using *tuf* as the reference, are reported as the mean ± SD of three biological replicates. Student's *t*‐test compared transcription in the wild type and in the variant. **p ≤ 0.01.

*V. vulnificus* cytotoxicity is contact‐independent (i.e., it does not require direct contact between bacteria and host cells), rather cytotoxic effector proteins are secreted into the extracellular milieu where they bind and enter target cells via surface receptors. This allowed us to examine cytotoxicity in a growth‐independent manner, whereby the supernatant of bacterial cultures grown in LBN to stationary phase (16 h growth, OD_600_ 2.5) was incubated with HeLa cells to evaluate lysis. In this experiment, the cell‐free supernatants of the two S522L mutants showed faster and significantly higher cytotoxicity than the wild‐type supernatants (Figure 6a). This suggested that during the pre‐incubation culture, the RpoB^S522L^ strains had produced and secreted a greater amount of cytotoxic proteins into the culture media. qRT‐PCR analysis of these pre‐incubation cultures, which had been grown in LBN to stationary phase, revealed significantly increased transcription of the *toxR*‐dependent *vvhA* hemolysin gene in the RpoB^S522L^ *V. vulnificus* compared to the wild type and a nonstatistically significant increase in the transcriptional levels of the *rpoS*‐dependent *rtxA* toxin gene, while no differences were observed for the *vvpE* elastase gene (Figure 6b).

## 4 DISCUSSION

Secondary effects of *rpoB* mutations conferring rifampicin resistance have been characterized in several bacterial species (Alifano et al., 2015; Billington et al., 1999; Cutugno et al., 2020; Jin & Gross, 1989; Jin et al., 1988a, 1988b; Wichelhaus et al., 2002). Evaluation of these effects is key to understanding how antibiotic resistance, already challenging per se, could affect the chances of a resistant microorganism surviving and spreading both in the environment and during infection (Gifford et al., 2018; Schenk & de Visser, 2013). Moreover, previously reported effects of rifampicin resistance on virulence traits of human pathogenic bacteria, show that such mutations can affect the success of the infection process (Björkman et al., 1998; Gao, et al., 2012). For these reasons, we investigated at phenotypic and transcriptional levels the effects of the S522L *rpoB* mutation in *V. vulnificus*.

We first analyzed the position of the RpoB residue S522 in the structural model of the *E. coli* RNAP, which has a high level of sequence conservation with the *V. vulnificus* enzyme. We observed that, although not close to the (p)ppGpp binding site, S522 is located in the active site region of the RNAP where mutations have the potential to alter the geometry of the site with possibly dramatic consequences for transcription. We demonstrated that the mutant strain growth defect is unique to growth in rich LBN media, while the growth rate is equal to or higher than the wild type in CDM.

Mutants with altered transcriptional machinery can exhibit a range of phenotypes (Alifano et al., 2015). In this work, we focused on the effects of the RpoB^S522L^ variant on the stress response, to provide data for predicting the potential survival and spread of the mutation in the population. Our previous report showed that the S522L mutants had a similar tolerance for high NaCl concentrations as the wild type (Cutugno et al., 2020). In contrast, here we showed a sensitivity of RpoB^S522L^ *V. vulnificus* to hypoosmotic stress and their inability to grow on MH media without NaCl supplementation. During these experiments there was visible growth of the wild type on both MH broth and agar, however, its viability was low after 24 h culture in MH broth. We hypothesize that the wild‐type bacteria underwent an initial period of replication, but that continued incubation in a hypoosmotic solution resulted in a reduction of cfu. In contrast, RpoB^S522L^ variants were unable to replicate in the hypoosmotic stress environment. As a possible cause of this unique stress survival profile, we observed increased transcription in the RpoB^S522L^ variants of the hyperosmotic stress‐related gene *putA* (Lee et al., 2003) and two major global regulators in *Vibrio* spp., *rpoS* and *toxR*. RpoS and ToxR control the expression of large regulons, with effects on growth, stress response, and virulence in many Gram‐negative human pathogens (Battesti et al., 2011; Dong & Schellhorn, 2010; Schellhorn, 2020; Skorupski & Taylor, 1997; Zhang et al., 2018). As increased transcription of these genes could therefore additionally impact the virulence of *V. vulnificus*, cytotoxicity of wild type and RpoB^S522L^ *V. vulnificus* was analyzed. While the mutants' growth defect in rich media was associated with delayed cytotoxicity for HeLa cells in a traditional bacteria‐eukaryotic cell co‐incubation experimental set‐up, RpoB^S522L^ *V. vulnificus* displayed strong contact‐independent cytotoxicity caused by the production and subsequent secretion of toxic molecules into the extracellular environment. This cytotoxic effect was significantly greater for the cell‐free supernatant of RpoB^S522L^ *V. vulnificus* precultures, compared to the wild type. Transcriptional analysis of the pre‐cultures (stationary phase bacteria grown in LBN) revealed significantly increased transcription of the *vvhA* hemolysin gene that is regulated by *toxR* (Guo et al., 2018; Lee et al., 2000). Regulation of transcription of *V. vulnificus* toxins is complex and involves several regulatory pathways (Choi & Choi, 2022; Elgaml & Miyoshi, 2017) which may in part explain the limited effect on *rtxA* and *vvpE* transcription in the variant compared to the wild type despite statistically significant increases in *rpoS* and *toxR* transcription. The temporal kinetics of toxin production in *V. vulnificus* should also be borne in mind. *rtxA* is maximally transcribed during the mid‐exponential phase (Park et al., 2012) *vvhA* during the mid‐late exponential phase (Choi et al., 2002) and *vvpE* during stationary phase (Jeong et al., 2001). When *V. vulnificus* was cultured in LBN at 30°C, VvhA was the most abundant and predominant extracellular protein and cytotoxin in stationary phase cultures (Hwang et al., 2011). In conclusion, this study demonstrates that *rpoB* mutations conferring rifampicin resistance can have dramatic effects on transcription in *V. vulnificus* with important consequences on its ability to survive in stressful environments and to infect the human host. In particular, the RpoB^S522L^ variant increases transcription of the stress‐response machinery with important implications for the likelihood of the strain surviving and spreading in the marine environment. Moreover, the same mutation causes increased production and secretion of cytotoxic molecules that confer a hypervirulent phenotype.

## AUTHOR CONTRIBUTIONS

**Laura Cutugno**: Conceptualization (equal); data curation (equal); formal analysis (lead); funding acquisition (equal); investigation (lead); methodology (equal); validation (lead); visualization (lead); writing—original draft (lead). **Conor O'Byrne**: Conceptualization (equal); funding acquisition (lead); methodology (equal); project administration (equal); writing—review and editing (equal). **Jan Pané‐Farré**: Conceptualization (equal); funding acquisition (equal); methodology (equal); supervision (equal); writing—review and editing (equal). **Aoife Boyd**: Conceptualization (equal); data curation (equal); funding acquisition (equal); methodology (equal); project administration (lead); supervision (lead); validation (equal); writing—review and editing (lead).

## CONFLICT OF INTEREST STATEMENT

None declared.

## ETHICS STATEMENT

None required.

## Data Availability

All data generated or analyzed during this study are included in this published article and its appendix.
